# Lactate in ruminant health: metabolic roles and the emerging significance of lactylation

**DOI:** 10.1186/s40104-026-01442-7

**Published:** 2026-06-29

**Authors:** Xiao Li, Maocheng Jiang, Yinghao Huang, Dian Wang, Jianbo Cheng

**Affiliations:** 1 College of Animal Science and Technology, Anhui Agricultural University, Hefei, 230036 China; 2 Anhui Province Key Laboratory of Local Livestock and Poultry, Genetical Resource Conservation and Germplasm Innovation, Anhui Agricultural University, Hefei, 230036 China; 3 National Center of Technology Innovation for Dairy, Hohhot, 010010 China

**Keywords:** Lactate, Lactylation, Metabolic, Ruminants

## Abstract

In ruminants, lactate metabolism is closely integrated with rumen fermentation, metabolic adaptation, and redox homeostasis under both physiological and pathological conditions. Under metabolic stress, alterations in lactate production, transport, and utilization may support tissue adaptation, but may also contribute to metabolic dysfunction and disease. As an emerging epigenetic modification, lactylation has been proposed as a mechanistic link between lactate metabolism and gene regulation, with lactate serving as a putative precursor. In this review, we summarize the dual roles of lactate in ruminant health and stress responses, evaluate the feasibility of lactate as a precursor for lactylation, and discuss the potential mechanisms and biological significance of lactylation in metabolic regulation and disease pathogenesis. Because direct evidence in ruminants remains limited, findings from non-ruminant models are considered only where they provide relevant mechanistic support. Overall, this review highlights current knowledge, major uncertainties, and key priorities for future mechanistic studies of lactate metabolism and lactylation in ruminants.

**Graphical Abstract:**

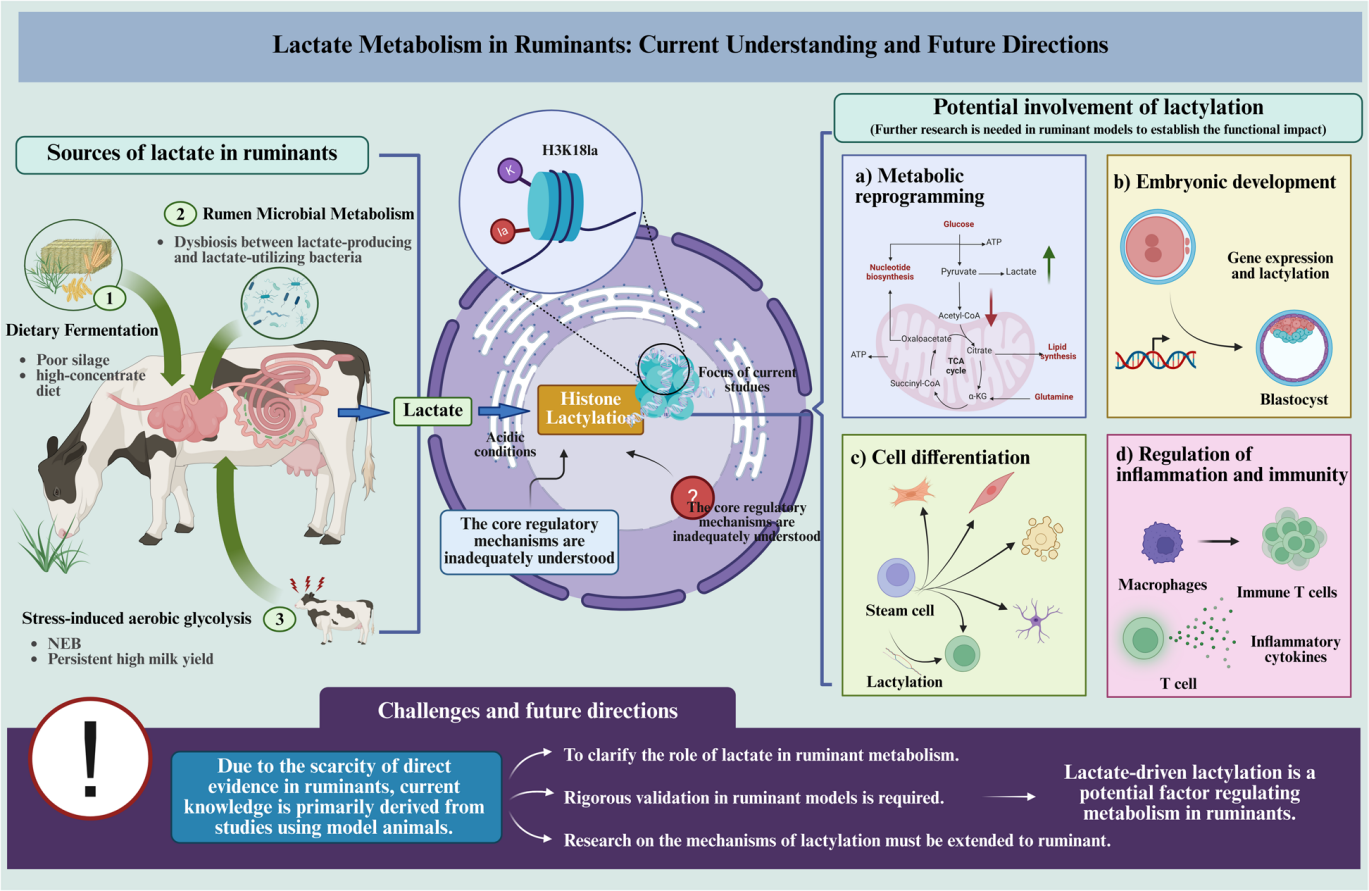

## Introduction

Epigenetic markers represent animal adaptations to environmental changes, enabling the acquisition of novel biological characteristics [1]. The markers eventually affect gene expression and ultimately affect the animal growth and health [2, 3]. Intermediate cellular metabolites constitute the chemical tags of these epigenetic markers [1]. Indeed, multiple studies have confirmed that animal organisms modify their metabolic states to adapt to complex environmental changes. The intricate interplay between metabolism and epigenetics has been increasingly substantiated, elucidating how alterations in animal physiology and environmental conditions influence gene regulation and phenotypic diversity [4–6]. Lactylation, an epigenetic process, is a novel type of epigenetic modification [7]. Lactate was previously wrongly regarded as a metabolic waste under low-oxygen conditions and was considered harmful [8]. Since the proposal of lactate formation and utilization under fully aerobic conditions [9], increasing evidence has shown that lactate functions as an important regulator of systemic metabolism and is increasingly recognized as a signaling molecule [10–14]. The identification of histone lysine lactylation established a molecular connection between cellular lactate levels and the epigenetic landscape. This epigenetic modification of metabolic regulation plays a crucial role in various biological stress. Various proteins in the cell nucleus, cytoplasm, mitochondria, endoplasmic reticulum, and cell membrane can undergo lactylation [15–21]. This indicates that lactylation may contribute to the regulation of biological processes, thereby opening new avenues for the study of metabolism-related diseases.

Reviews addressing lactylation in ruminants remain limited. This article summarizes the mechanisms of lactate metabolism in ruminants, the effects of lactate on ruminant health, and the feasibility of metabolically derived lactate serving as a precursor for lactylation. It also highlights the potential significance of lactate and lactylation in ruminant production, biological function, and health.

## Lactate metabolism and its distinctive features in ruminants

### Overview of lactate metabolism

**Fig. 1:**
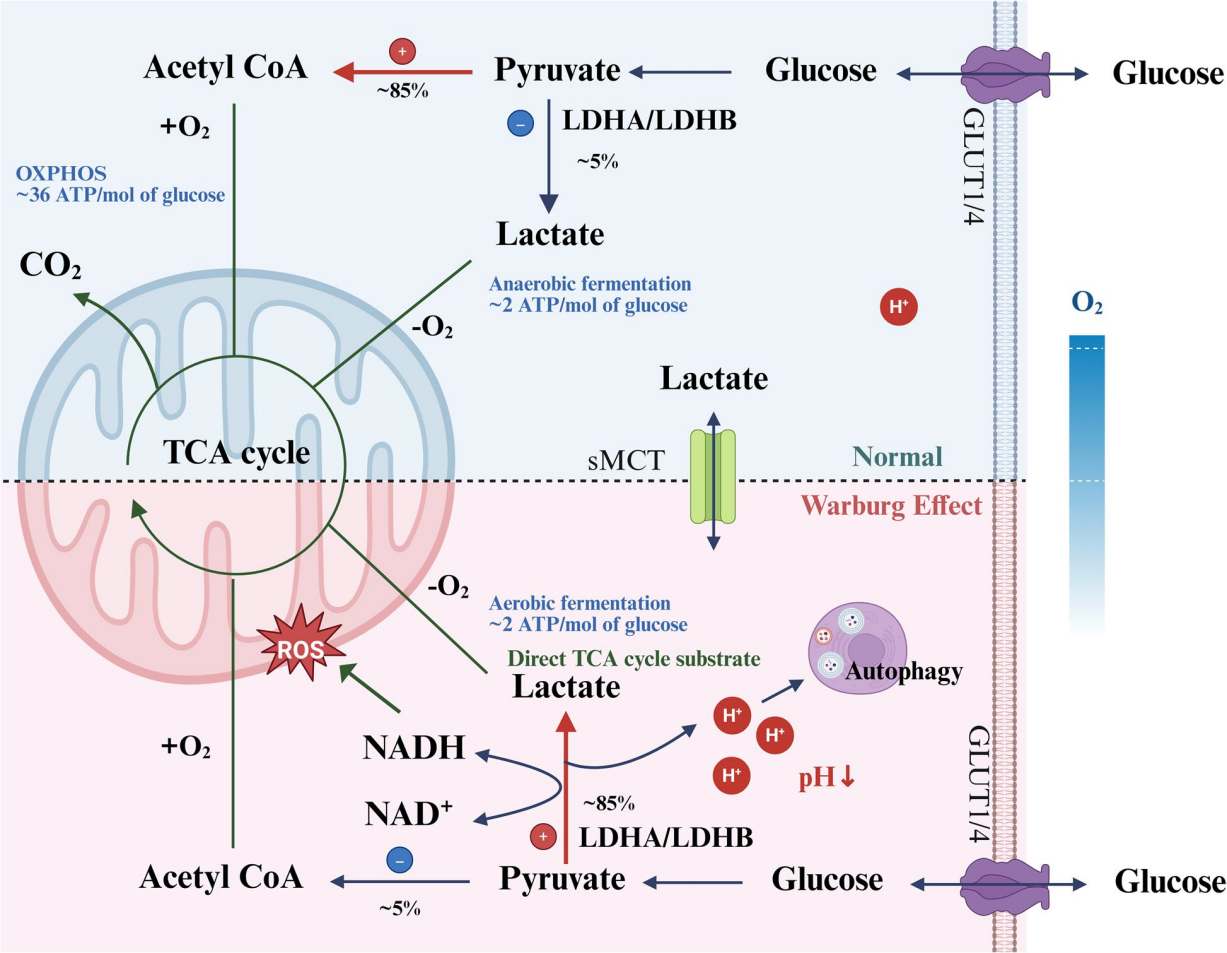
The overview of aberrant lactate-related metabolism compared to physical conditions. When cellular demand for oxygen and ATP exceeds supply, glycolytic flux is enhanced to meet increased energy and metabolic demands. This shift increases glucose consumption, promotes excessive cytosolic lactate production, and reduces ATP yield. Under physiological conditions, pyruvate, the end product of glycolysis, enters the mitochondria and is oxidized through the tricarboxylic acid (TCA) cycle to generate energy. By contrast, in cells exhibiting the Warburg effect, pyruvate is preferentially converted to lactate rather than entering the TCA cycle, resulting in less efficient ATP production per molecule of glucose. Under hypoxic conditions, lactate plays a critical role in regenerating NAD^+^, thereby sustaining glycolysis and ATP production. Meanwhile, the accumulation of NADH and H^+^ contributes to oxidative stress and autophagy. Because evidence for the Warburg effect in ruminant cell models remains limited, this figure was compiled from available data in ruminant and non-ruminant systems. Abbreviations: *MCT* Monocarboxylate transporter, *GLUT1/4* Glucose transporter, *TCA* Tricarboxylic acid, *OXPHOS* Oxidative phosphorylation, *LDHA/B* Lactate dehydrogenase A/B

Nearly all animals derive energy through two primary pathways: anaerobic fermentation and oxidative phosphorylation [22, 23]. Lactate serves as the principal product of both pathways [22, 24, 25] and functions simultaneously as a key metabolic substrate [26] and an essential signaling molecule [10]. The phenomenon whereby glucose is converted to lactate under aerobic conditions, instead of undergoing mitochondrial oxidation, is known as the "Warburg effect" [27]. This effect was initially observed in cancer cells [28]. Emerging evidence indicates that most mammalian cells are capable of producing lactate even in the presence of sufficient oxygen [26]. Moreover, aerobic glycolysis has been shown to occur persistently under various stress conditions, including exercise, high-altitude exposure, trauma, pancreatitis, sepsis, myocardial infarction, and heart failure [23]. The pronounced activity of aerobic glycolysis is primarily driven by an increased cellular demand for ATP [29], with the most immediate consequence being elevated lactate concentrations both intracellularly and extracellularly [30]. This process is schematically illustrated in Fig. 1, which depicts lactate metabolism under both normal and pathological conditions.

Lactate exists as two stereoisomers, L-lactate and D-lactate, with L-lactate being the predominant product of anaerobic fermentation in mammals [31]. In contrast, the intestinal microbiota generates a mixture of both L- and D-lactate enantiomers [15]. L-Lactate is efficiently digested and absorbed in the intestines and other tissues, whereas D-lactate typically accumulates within the intestinal lumen [15]. The physiological effects of lactate depend on an efficient lactate shuttle system. The lactate shuttle comprises the transport and intercellular communication processes through which lactate acts as an oxidative substrate, an inflammatory modulator, and a signaling molecule across cells, tissues, and organ systems [9, 15, 32–34]. A representative example is the "intestinal–systemic lactate shuttle", in which lactate generated by microbial fermentation enters the circulation via sodium-coupled monocarboxylate transporters (sMCTs) and exerts diverse physiological functions [35].

Lactate metabolism is broadly involved in physiological regulation and metabolic adaptation, including hypoxic stress responses [36], autophagy [37], embryonic development [38], immunity [39], and aging [40]. By contrast, pathological conditions are often accompanied by lactate accumulation and lactate shuttle dysfunction [41], and in animal production systems, elevated lactate is closely linked to stress severity [42–44] and acidosis [45]. Thus, the biological effects of lactate are strongly shaped by metabolic state, cellular context, and disease condition [46]. Emerging evidence suggests that lactate may participate in epigenetic regulation through lactylation [47]. However, direct support for this mechanism in ruminant models remains scarce.

### Distinctive features of lactate metabolism in ruminants

Lactate serves as an important energy substrate for dairy cows [48]. In the rumen, lactate originates from exogenous feed [49] as well as endogenous microbial synthesis [50, 51]. Single-cell transcriptomic analyses have revealed high metabolic activity among rumen microorganisms involved in carbohydrate metabolism in dairy cows [52]. These rumen microbes contribute to lactate metabolism [53] primarily through glucose fermentation [49], encompassing both lactate-producing bacteria, such as *Streptococcus bovis* and *Lactobacillus* spp. [54], and lactate-utilizing bacteria, including *Megasphaera elsdenii* [55] and *Selenomonas ruminantium* [56].

**Fig. 2:**
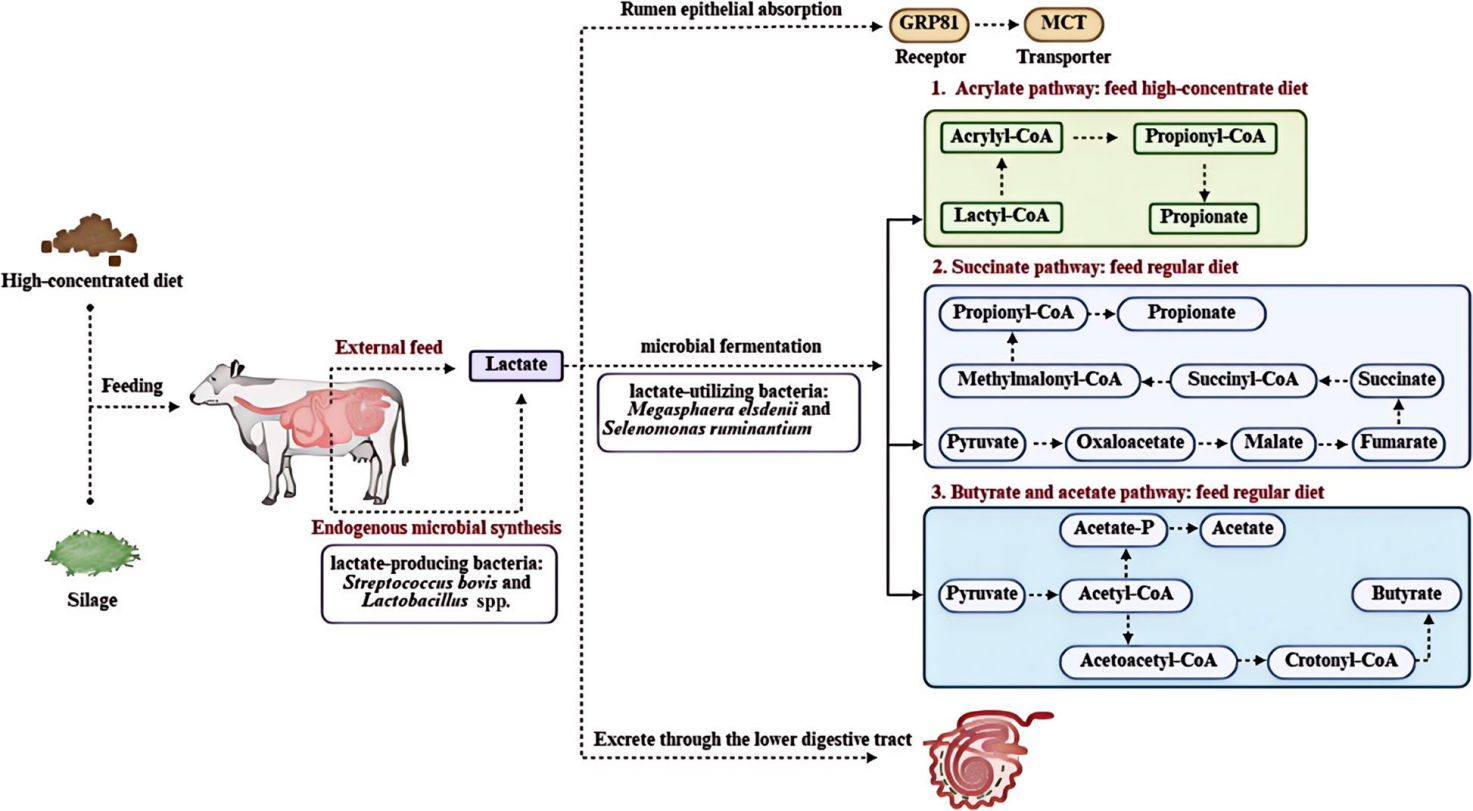
Lactate metabolism in ruminants. In ruminants, lactate originates mainly from high-concentrate diets, poor-quality silage, and ruminal lactate-producing bacteria. Its major fates include absorption by the ruminal epithelium, microbial fermentation, and excretion through the lower digestive tract

In dairy cows, ruminal lactate is metabolized mainly through the succinate, acetic–butyric acid, and acrylate pathways [57]. Under conventional feeding conditions, the succinate and acetic–butyric acid pathways predominate [57–59], with lactate first being converted to pyruvate by lactate dehydrogenase and then further metabolized to downstream organic acids [60]. Under high-concentrate feeding, lactate metabolism shifts mainly to the acrylate pathway [57–59]. Because D-lactate is utilized less efficiently than L-lactate in mammalian systems [61], it is first converted to L-lactate by lactate racemase before entering subsequent metabolism [62]. These pathways are illustrated in Fig. 2.

Besides being further fermented into volatile fatty acids, lactate derived from rumen microbial metabolism can also be absorbed by ruminal epithelial cells [63]. In the rumen, lactate functions both as a signaling molecule through GPR81 [64] and as a transport substrate for monocarboxylate transporters (MCT), particularly MCT1, MCT2, and MCT4 [65–67]. Among these, MCT1 plays a central role in lactate transport and in the regulation of anaerobic fermentation and oxidative metabolism in ruminal epithelial cells [66, 68, 69]. Moreover, sMCTs contribute to lactate uptake from the rumen, whereas inhibition of MCT1 impairs lactate transport and promotes the accumulation of monocarboxylic acids in the rumen [70].

Under metabolic stress, the coordinated lactate homeostasis maintained by the rumen microbiota and epithelium is disrupted. This systemic metabolic reprogramming, involving hepatic gluconeogenesis, lipid oxidation, and ketogenesis, impairs lactate turnover and promotes its pathological accumulation.

## Dual roles of lactate in ruminant health and disease

### Lactate accumulation and major metabolic disorders

#### Disorders associated with ruminal acidosis

In production systems, ruminants are commonly fed high-concentrate diets to support high milk yield [71, 72]. Excessive carbohydrate intake disrupts the balance between lactate-producing and lactate-utilizing bacteria, thereby promoting lactate accumulation and contributing directly to the development of ruminal acidosis [73, 74]. Available evidence indicates that rumen protozoa are important regulators of lactate metabolism [75]. Under faunated conditions, lactate is rapidly utilized and mainly converted to propionate, whereas defaunation slows lactate disappearance and shifts fermentation toward butyrate production [75]. In vitro studies further support a major role for protozoa in ruminal lactate utilization, as the protozoal fraction shows a much higher lactate disappearance rate than the bacterial fraction [75]. Moreover, excessive lactate production resulting from improper silage use in livestock production can disrupt rumen fermentation [76].

Elevated ruminal lactate can disrupt energy metabolism and promote inflammation in ruminants by altering the rumen microbiota [77]. High D-lactate conditions are also associated with an increased abundance of *Escherichia-Shigella*, suggesting a greater risk of gastrointestinal infection [77]. In lactating goats, grain-induced subacute ruminal acidosis (SARA) has been shown to increase epithelial permeability and impair barrier function [78]. More broadly, SARA disrupts the rumen microbiota and promotes the production of toxic metabolites, including lipopolysaccharides (LPS) and lactate [79, 80].

Lactate, together with LPS, has been identified as a contributor to mastitis in dairy cows [81]. In cases of SARA, LPS originating from the rumen can translocate to the mammary gland via systemic circulation, triggering inflammatory responses [82, 83]. Lactate can exacerbate inflammation during chronic inflammatory conditions [84], and LPS modulates lactate-induced inflammatory responses in the bovine mammary gland through the hypoxia-inducible factor-1α (HIF-1α)/MCT1 pathway [49, 85, 86]. Interestingly, in non-ruminant animal models, lactate inhibit the inflammatory factors in acute inflammatory diseases [87], tumors [88], and immune cells [89]. The dualistic pro- and anti-inflammatory effects of lactate are determined by several factors, including its chirality, the responding cell types, and the specific local microenvironmental conditions [22].

In ruminants, the lack of efficient D-lactate-metabolizing enzymes results in much slower mitochondrial clearance of rumen-derived D-lactate than of L-lactate [90]. Consequently, D-lactate readily accumulates in the circulation and becomes a major contributor to metabolic acidosis [91]. During acute ruminal acidosis (ARA), blood D-lactate can rapidly approach 5 mM and is associated with systemic lesions, including rumenitis, liver abscesses, polioencephalomalacia, particularly in calves, and lameness-related disorders [90–93]. In transition dairy cows, increased ruminal D-lactate production is directly associated with clinical acidosis, multiple-joint swelling, and aggravated lameness [94]. Likewise, synovial fluid metabolomics in heifers with ARA has revealed marked increases in D-lactate and L-lactate, together with reprogramming of starch and sucrose metabolism, pyruvate metabolism, and glycolysis [95]. These findings support a close link between D-lactate accumulation and bovine laminitis and polysynovitis [96, 97]. Causal evidence is provided by intervention studies. Intraruminal infusion of lactate induces laminitis in sheep [98], whereas excessive oligofructose intake in dairy cows triggers systemic aseptic neutrophilic polysynovitis that, together with laminitis, contributes to lameness during ARA [99]. Temporal studies further show that lactate elevation precedes the onset of lameness in fructooligosaccharide-induced ARA [100], and that experimental ARA in sheep causes an early surge in blood D-lactate accompanied by increased acute-phase proteins, including haptoglobin and ceruloplasmin [101]. Collectively, these observations highlight the strong association between D-lactate and inflammatory pathology [96]. Mechanistically, D-lactate exerts pro-inflammatory effects through direct activation of immune cells. In bovine polymorphonuclear leukocytes (PMNs), D-lactate increases CD11b expression [102], promotes adhesion to endothelial cells [103], and stimulates neutrophil extracellular trap (NET) formation at concentrations relevant to ARA [103]. These effects depend on D-lactate uptake through MCT1, as MCT1 inhibition markedly attenuates them [103].

Neonatal diarrhea is a major health concern in calves, with metabolic acidosis as its principal complication [104, 105]. Elevated concentrations of both D-lactate and L-lactate have been detected in diarrheic calves, and increased D-lactate levels in serum and feces further indicate that dysregulated intestinal bacterial fermentation is a key driver of diarrheic acidosis [106]. Consequently, D-lactate accumulation is considered a major contributor not only to acidosis [106] but also to central nervous system impairment in affected neonatal calves [46, 107]. In young ruminants, acidosis may also result from esophageal groove dysfunction, which causes milk to enter the rumen, and under such conditions D-lactate may further promote inflammation [108, 109].

In conclusion, ruminants frequently develop D-lactate as a result of disrupted digestion and fermentation caused by excessive intake of grains and carbohydrates or by infectious diarrhea [110–112].

#### Negative energy balance and lactate accumulation

The periparturient period is a critical stage in dairy cows, characterized by nutrient partitioning toward fetal development, udder growth, and subsequent lactation. During this period, reduced dry matter intake (DMI) before calving leads to negative energy and protein balance [113], forcing cows to mobilize fat and muscle reserves to meet increasing metabolic demands [114].

During negative energy balance (NEB) with insufficient glucose supply, obligatory glucose consumption by peripheral tissues (e.g., muscle) for basal functions indirectly limits mammary glucose availability, thereby constraining lactose synthesis despite the mammary gland’s high metabolic priority [115]. Dairy cows begin mobilizing muscle reserves prior to calving and continue this process for approximately 30 to 60 DIM [116–118]. Excessive mobilization of the organization is often associated with adverse events [119]. Muscle turnover, defined as the balance between protein synthesis and degradation [120], occurs in muscles composed of three main fiber types: type I, IIA, and IIX [121]. Glycolytic muscle fibers, such as type IIX fibers, rely more heavily on glucose due to their use of anaerobic metabolic pathways during contraction [121]. Evidence indicates that, during the prenatal and postnatal periods, bovine muscle fibers shift from type IIA to type IIX [115], This transition suggests that the onset of lactation may enhance glycolytic activity, potentially leading to increased lactate accumulation in the body.

Adipose tissue is the primary energy reservoir in dairy cows and undergoes extensive lipolysis during early lactation as a key adaptation to periparturient NEB [122]. This process reduces fat reserves, alters adipokine secretion, and increases the use of non-esterified fatty acids (NEFAs) to support maintenance and lactation [123]. However, sustained and excessive lipolysis elevates circulating NEFA concentrations, thereby promoting inflammation and metabolic dysfunction. To compensate for limited glucose availability, dairy cows further enhance fat mobilization, but this response progressively impairs insulin action and disrupts insulin signaling, ultimately leading to insulin resistance (IR) [124–126].

IR affects multiple insulin-sensitive tissues. In skeletal muscle, it impairs glucose transport and utilization, reduces glycogen synthesis, and shifts energy metabolism toward greater fatty acid oxidation, thereby increasing NEFA release [127]. In adipose tissue, IR dysregulates lipolysis and further elevates circulating NEFA levels [124]. In the liver, IR is characterized by impaired insulin-mediated suppression of gluconeogenesis, whereas its stimulatory effect on de novo lipogenesis is relatively preserved [128]. Although this pattern might suggest enhanced lactate utilization, ketotic cows under NEB show elevated blood concentrations of both lactate and lactate dehydrogenase [43, 129]. This apparent paradox in the insulin-resistant liver may be mechanistically linked to excess NEFAs.

Following parturition, enhanced adipose mobilization in dairy cows leads to a substantial influx of NEFAs into the liver [130]. These NEFAs are mainly catabolized through β-oxidation, generating large amounts of acetyl-CoA for ketone body production [131]. Under NEB, excess NEFAs and β-oxidation-derived acetyl-CoA inhibit pyruvate dehydrogenase (PDH), thereby restricting pyruvate entry into the tricarboxylic acid (TCA) cycle [132]. As a result, pyruvate is less efficiently oxidized and is more readily diverted toward lactate production. In parallel, dairy cows activate a glucose-sparing mechanism that prioritizes glucose supply to the mammary gland for lactose synthesis [133, 134]. This shift further limits the role of glucose as a systemic energy substrate, constrains TCA cycle activity, and enhances anaerobic glycolysis, ultimately promoting lactate accumulation [135]. Collectively, these mechanisms help explain the elevated circulating lactate concentrations observed in dairy cows with severe negative energy balance.

#### Oxidative stress and mammary health

Mastitis, ruminal acidosis, NEB, metritis, and severe heat or hypoxic stress can all induce oxidative stress in dairy cows [136, 137]. Beyond directly causing tissue injury, oxidative stress also acts as an upstream signal that reprograms energy metabolism and promotes lactate accumulation [138]. Under these conditions, dairy cows are prone to metabolic hypoxia, which shifts cellular metabolism from aerobic oxidation toward anaerobic fermentation [139, 140]. Excess mitochondrial ROS further enhances LDHA-mediated conversion of pyruvate to lactate [141]. However, evidence from pancreatic cancer cells indicates that MCT1-dependent lactate uptake can also facilitate ROS neutralization [142]. Direct evidence for this specific mechanism in ruminant models scarce. Thus, lactate may both suppress anaerobic glycolysis through product feedback inhibition [143] and serve as an oxidative substrate that mitigates oxidative stress by eliciting a mild ROS burst [144, 145]. Collectively, these findings suggest that the relationship between oxidative stress and lactate is bidirectional and highly context dependent, being shaped by lactate concentration, exposure duration, and cell type.

During lactation, dairy cows undergo pronounced hormonal fluctuations, while colostrum secretion after calving and sustained high milk yield impose a substantial oxygen demand, thereby promoting reactive oxygen species (ROS) accumulation and oxidative stress [146, 147]. Consistently, during peak lactation, the bovine mammary gland exhibits markedly elevated oxygen consumption, and mammary oxidative stress is greater than that observed in later lactation [148]. Under oxidative stress and metabolic hypoxia, lactate is generated as a key metabolite that diverts pyruvate flux away from the TCA cycle toward fermentation, while together with pyruvate contributing to redox homeostasis [26]. In line with this view, the mammary glands of high-yielding dairy goats show higher ROS levels and more active lactate metabolism than those of low-yielding animals [149, 150].

Furthermore, insufficient glucose supply in early-lactation dairy cows markedly upregulates HIF-1α expression in the mammary gland, indicating hypoxic stress [151]. More broadly, glucose metabolic reprogramming appears to be a key adaptive response to stress in ruminants, with lactate metabolism at its core [152]. Similar evidence has been reported in yak testicular Sertoli cells, in which hypoxia enhances glucose metabolism and increases lactate production and transport [152]. Taken together, these findings suggest that limited glucose availability promotes lactate accumulation through hypoxia-driven metabolic reprogramming. In response, dairy cows mobilize adipose reserves, and approximately half of the NEFAs released during lipolysis are taken up by mammary epithelial cells [153, 154]. Elevated NEFA concentrations then exert lipotoxic effects, enhance ROS generation, and aggravate redox imbalance and mitochondrial dysfunction in the bovine mammary epithelium [155]. Conversely, excessive glucose supply can also induce hypoxic stress in the mammary gland, leading to ROS accumulation and mammary epithelial cell apoptosis [156]. In addition, heat stress has been shown to promote lactate accumulation in dairy cows [157]. Thus, lactate homeostasis is a critical determinant of lactation performance and dry matter intake, and maintaining this balance is essential for sustaining high milk yield [156]. Notably, excessive lactate can impair mitochondrial quality control [158] and may further induce mammary oxidative stress and circadian disruption through HIF-1α stabilization, ultimately reducing milk production [159].

### Potential beneficial effects of lactate

Although lactate accumulation is often associated with metabolic disorders, lactate itself is a central endogenous metabolite that can exert beneficial effects under physiological concentrations and in specific microenvironments, particularly in energy homeostasis and reproductive development. As an efficient energy carrier and gluconeogenic precursor, lactate may improve metabolic balance in ruminants. Dietary lactate supplementation has been suggested to enhance energy balance in dairy cattle [160], possibly because exogenous lactate is preferentially utilized by rumen microorganisms such as *Megasphaera elsdenii*, thereby stabilizing ruminal pH and redirecting fermentation toward glucogenic volatile fatty acids, especially propionate [161]. Because propionate is the most efficient precursor for hepatic gluconeogenesis in ruminants [162], lactate may enhance glucose production through the rumen–liver axis and thus support energy-demanding processes such as lactation. In immature lambs, lactate-derived glucose via the Cori cycle can account for up to 49% of total glucose production [162]. In addition, circulating lactate can be redistributed through the lactate shuttle, providing an efficient means of intercellular energy transfer [163]. In ruminants, the mammary gland is a highly energy-demanding organ and can utilize circulating lactate as a metabolic substrate [164], although the lactate shuttle remains insufficiently characterized in these species.

At appropriate concentrations, lactate is an important component of the microenvironment required for germ cell function and early embryonic development. In 8-cell somatic cell nuclear transfer (SCNT) embryos, weighted gene co-expression network analysis identified L-LDHA as a hub gene, while both LDHA expression and lactate levels were lower than in in vitro fertilization embryos [165]. Consistently, lactate supplementation improves SCNT embryo developmental competence and blastocyst quality [165]. In early goat embryos, lactate accumulated under physiological oxygen conditions regulates development by inducing histone H3K18 lactylation and upregulating the m6A methyltransferase METTL3 [166]. Similarly, high lactate concentrations have been detected in the follicular fluid of buffaloes and sheep, where the balance among lactate, glucose, and pyruvate exerts cell type-specific effects on oocytes and granulosa cells [167]. In bovine granulosa cells, L-lactate suppresses CYP19A1, downregulates FSHR and LHCGR, and upregulates early luteinization markers; these effects are abolished by PKA inhibition, indicating that lactate promotes granulosa cell differentiation during the follicular-to-luteal transition [168]. The negative and positive effects of lactate in ruminants are summarized in Table 1.

**Table 1:** Effects of lactate accumulation in ruminants

Item	Motivation	The reason for lactate accumulation	Disease	Influence	Reference
Negative research	High-concentration diets	Metabolic disorder of ruminal microorganisms	Ruminal acidosis	Gastrointestinal infections, bovine laminitis, polysynovitis and mammitis	[49, 77, 82, 83, 85, 95–97]
	Improper utilization of silage	Excessive intake of lactate			[76]
	Milk entering the rumen or improper use of feed	Poor fermentation of intestinal bacteria	Calf diarrhea	Central nervous system impairment	[46, 106, 107]
	Negative energy balance	Most of the glucose is allocated to the mammary glands for lactose synthesis. The energy supply function of glucose is weakened, while anaerobic glycolysis is strengthened	Ketosis	Production performance decline and inflammation	[169–171]
	Oxidative stress	Metabolic hypoxia causes the cellular energy metabolism to shift from aerobic oxidation to anaerobic glycolysis	Mastitis, ruminal acidosis, NEB, metritis, as well as severe heat stress and hypoxic stress	Production performance decline and inflammation	[26, 136, 152, 155, 158, 159, 172]
Positive research	The inclusion of lactate in dairy cattle feed promotes energy balance				[160]
	Supplementation of lactate enhance the developmental potential and embryo quality of bovine SCNT				[165]
	Lactate can induce the differentiation of bovine granulosa cells				[168]

## Stress-induced lactate metabolic reprogramming in ruminants

As discussed above, lactate serves as both an energy substrate and a signaling molecule in the regulation of diverse cellular processes [173]. Mechanistically, its effects are mediated by extracellular sensing through GPR81 and intracellular actions after transport via monocarboxylate transporters [174].

GPR81, also known as HCA1, is the principal G protein-coupled receptor activated by lactate [175]. Plasma lactate concentrations reported in exercised dairy cows [176], cows with abomasal disorders [177], and acidotic cows [91, 178] are sufficient to activate this receptor. In ruminants, GPR81 expression has been reported to increase in retroperitoneal adipose tissue during the first 21 days postpartum [179]. By contrast, a high-energy diet downregulates GPR81 expression in mesenteric, omental, and subcutaneous adipose tissue of non-pregnant, non-lactating dairy cows [180], although this effect has not been consistently observed in high-yielding dairy cows [179]. These findings suggest that GPR81 regulation is context dependent. Functionally, extracellular lactate activates GPR81/HCA1, inhibits adenylate cyclase, lowers intracellular cAMP, and thereby modulates PKA activity and cellular metabolism [181]. However, the abundance of GPR81 in ruminants, and the extent to which lactate signals through this receptor, remain incompletely defined [175]. Moreover, lactate can also exert GPR81-independent effects, including modulation of LPS-induced macrophage activation [182] and induction of the neutrophil chemokines CXCL1 and CXCL2, as well as granulocyte colony-stimulating factor (G-CSF) [183]. These receptor-independent actions may depend, at least in part, on lactate transporters.

In ruminants, MCT-mediated lactate transport has been well documented [67, 68, 184]. Several MCT subtypes have been identified in bovine rumen epithelial cells [66] and neutrophils [103], with MCT1 and MCT4 being the most extensively studied in the rumen [67, 68, 184]. MCT1 is localized mainly to the basolateral membrane of the ruminal epithelium and mediates lactate efflux into the bloodstream [185]. In contrast, MCT4 and SMCT1 are located primarily on the apical membrane and mediate lactate uptake from the rumen lumen into epithelial cells [186]. This distribution is consistent with the changes in lactate absorption observed during high-grain feeding [187, 188] and with the extremely low apical MCT activity reported in roughage-fed sheep and goats [48]. Collectively, these findings support the view that lactate accumulation in ruminants is not merely a metabolic consequence, but also a signaling event mediated through both GPR81-dependent sensing and MCT-dependent intracellular pathways.

Evidence from porcine liver [189] and rumen epithelial cells [190] indicates that MCT1 expression is regulated by peroxisome proliferator-activated receptors (PPARs). Inhibition of MCT1 markedly reduces intracellular lactate levels and suppresses inflammatory cytokine expression [97]. By contrast, MCT4 appears to be regulated, at least in part, by HIF-1 [191–193], which is itself influenced by PPAR signaling [194]. Collectively, these findings link lactate-mediated stress responses to pathways involved in energy metabolism, inflammation, and hypoxic adaptation.

Consistent with this view, L-lactate is associated with both high milk production and increased ROS accumulation [159]. In bovine mammary epithelial cells (BMECs), sodium L-lactate levels are positively correlated with HIF-1α expression and oxidative stress, whereas HIF-1α knockdown attenuates oxidative stress [159]. Likewise, inhibition of HIF-1α reduces LDH expression and lactate production, although lactate can also induce ferroptosis in yak muscle cells independently of HIF-1α [195]. Conversely, oxidative stress further promotes lactate accumulation. In yak longissimus dorsi muscle, ROS-induced oxidative stress enhances lactate accumulation through activation of the CaMKKβ/AMPK pathway [196]. Similarly, in yak testicular cells, hypoxia promotes lactate production and glucose metabolic reprogramming by driving the degradation of GLUT3, GLUT8, and MCT4 [152].

Moreover, lactate, particularly D-lactate, is a critical mediator linking metabolic disturbances in ruminants to inflammatory responses [197, 198]. Consistently, experimental ruminal acidosis induced by oligofructose overload increases PMNs infiltration and elevates inflammatory mediators, including IL-6, IL-8, and PGE2, in synovial fluid [198]. Mechanistically, D-lactate promotes metabolic reprogramming in bovine PMNs through mtROS, PI3K/Akt/HIF-1-, and GSK-3β-dependent pathways, thereby facilitating extracellular trap (ET) formation [109, 199]. In line with this, inhibition of mitochondrial complex I or scavenging of mtROS attenuates D-lactate-induced ET formation [97]. In addition, D-lactate enters bovine fibroblast-like synoviocytes (bFLS) via MCT1 and activates the MAPK, PI3K/Akt, and NF-κB pathways, thereby promoting IL-6 and IL-8 production [97]. Because PI3K/Akt regulates NF-κB nuclear translocation [200, 201], and is closely linked to HIF-1α regulation [202], D-lactate-induced HIF-1α accumulation appears to be a central event in this response. Indeed, inhibition of HIF-1α reverses the D-lactate-induced upregulation of HIF-1α, GLUT1, PDK1, LDHA, and IL-6, indicating that HIF-1α plays a pivotal role in mediating D-lactate-driven metabolic reprogramming and inflammatory activation in bFLS under normoxic conditions [97].

**Fig. 3:**
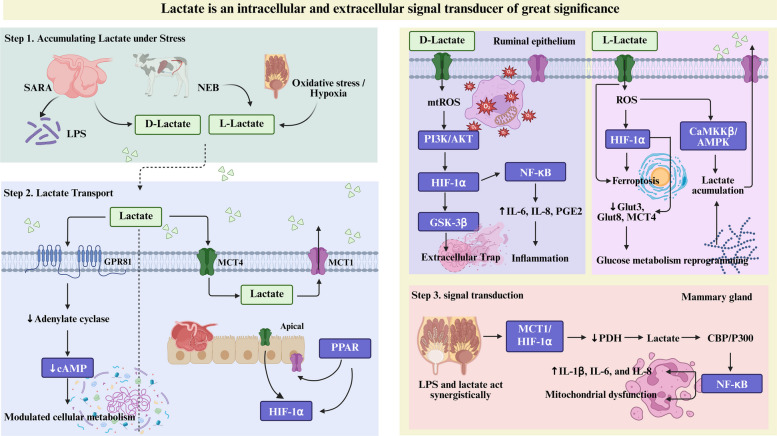
Lactate is an intracellular and extracellular signal transducer of great significance. Lactate affects the expression of inflammatory and oxidative stress-related genes in ruminant cells through PI3K/AKT, NF-κB, HIF-1α and their related pathways. Lactate does not only play a role inside the cell. Outside the cell, GPR81 transports lactate and promotes the utilization of lactate through subsequent signal transduction. Abbreviations: *cAMP* Cyclic adenosine monophosphate, *CaMKKβ/AMPK* Calcium/calmodulin-dependent protein kinase kinaseβ/adenosine 5′-monophosphate (AMP)-activated protein kinase, *HIF-1α* Hypoxia inducible factor-1α, *IL* Interleukin, *GLUT* Glucose transporter, *GPR81* G-protein-coupled receptor 81, *mtROS* Mitochondria reactive oxygen species, *MCT1/4* Monocarboxylate transporter 1/4, *NF-κB* Nuclear factor kappa-B, *PI3K/AKT* Phosphoinositide 3-kinase/protein kinase B

Moreover, LPS generated during ruminal dysbiosis may act synergistically with lactate to amplify inflammatory responses. Consistently, high-concentrate feeding promotes ruminal lactate accumulation [48] and increases LPS levels in caprine blood [203], as well as in bovine rumen fluid and mammary arterial and venous blood [204]. In BMECs, LPS increases lactate production by reprogramming glycolysis through the HIF-1α/MCT1 pathway while suppressing PDH activity [85]. Because the balance between anaerobic glycolytic flux and PDH-mediated pyruvate oxidation is a key determinant of lactate levels [205], this shift further favors lactate accumulation. Lactate, in turn, exacerbates inflammation by upregulating IL-1β, IL-6, and IL-8 expression and enhancing NF-κB activation through P300/CBP-dependent mechanisms [49]. In the mammary gland, lactate activates MAPK signaling and promotes IL-6 and IL-8 expression [206]. It also suppresses mitochondrial biogenesis regulators, including SIRT1, PGC-1α, NRF1, and TFAM, while increasing adenine nucleotide translocases, VDAC1, and cyclophilin D, thereby contributing to mitochondrial dysfunction and mammary injury in dairy cows [206]. Figure 3 presents a core signaling transduction framework for lactate.

Nevertheless, the mechanisms by which lactate engages these signaling pathways remain poorly defined, especially in epigenetic regulation. Evidence that D-lactate induces post-transcriptional accumulation of HIF-1α in bovine PMNs, together with the close association between lactate abundance and protein lactylation, suggests that lactate may reshape gene transcription through lactylation and thereby influence ruminant physiology [47, 70, 97, 207].

## Lactate-driven lactylation in ruminants

### From lactate metabolism to lactylation

Lysine lactylation (Kla) is an emerging mechanism by which lactate links metabolism to epigenetic regulation [47]. In the cytoplasm, lactate drives non-histone lactylation and modulates protein activity [20, 21]. In the nucleus, it promotes histone lactylation, weakens histone-DNA interactions, and enhances promoter-associated transcription [47, 208, 209].

**Fig. 4:**
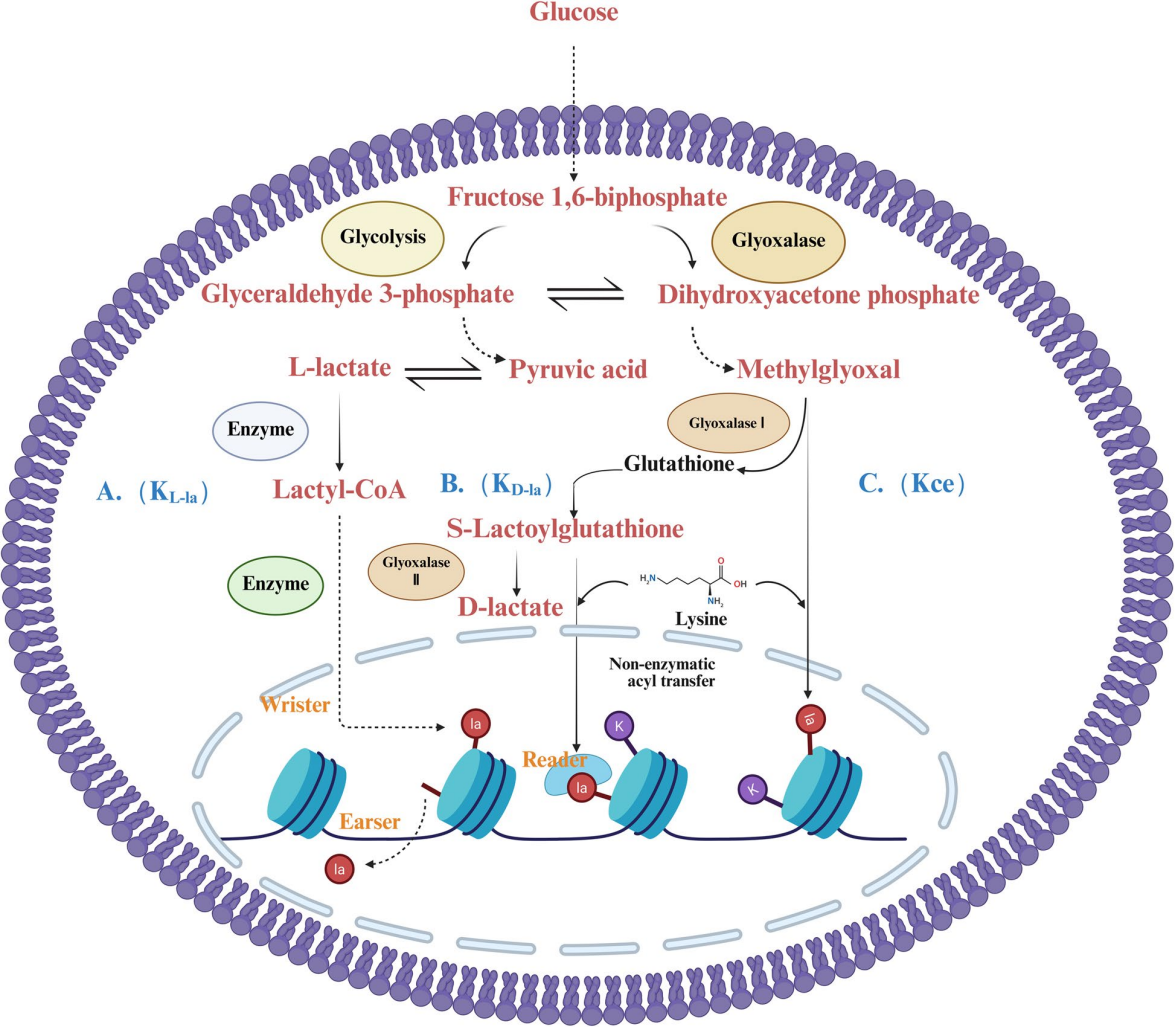
Possible patterns of lactylation. Lysine L-lactylation (K_L-la_) is a recently identified protein post-translational modification (PTM) driven by L-lactate. Lactylation-related modifications include three major forms: K_L-la_, Nε-(carboxyethyl)lysine (K_ce_), and D-lactyl-lysine (K_D-la_), with K_L-la_ considered the principal form associated with fermentation and the Warburg effect. Because evidence regarding the enzymatic and non-enzymatic mechanisms of lactylation in ruminants remains limited, this figure was constructed primarily on the basis of findings from non-ruminant models

Lactylation is thought to occur through three principal mechanisms (Fig. 4). The first is enzyme-mediated histone L-lactylation(K_L-la_), in which lactyl-CoA serves as the donor substrate and writer, eraser, and reader enzymes cooperatively generate K_L-la_ [21, 47, 210, 211]. The second is non-enzymatic D-lactoylation (K_D-la_), which uses LGSH as the donor substrate and may also occur on metabolic enzymes [212]. In this process, Glyoxalase II (GLO2) regulates lysine lactoylation by S-lactoylglutathione (LGSH) turnover and glutathione regeneration. The third is carboxyethylation, in which methylglyoxal (MGO) modifies cysteine, arginine, and lysine residues to form Nε-(carboxyethyl)lysine (Kce) [213]. Table 2 summarizes the key debates and unknowns in lactylation research.

**Table 2:** Controversies in lactylation modification

Controversial issues	Supporting evidence	Reasons for the controversy
Are the mechanisms of lactylation mediated by the two lactate stereoisomers identical?	Some studies propose that L-lactyl-CoA corresponds to enzymatic L-lactylation, whereas S-D-lactoylglutathione (SLG) corresponds to non-enzymatic D-lactylation [7, 214]	Many studies may have conflated distinct precursors, such that cross-study comparisons may not in fact be addressing the same lactylation PTM
Is lactylation a "major driver" or a "minor regulator"?	Many studies supporting a "driver" role have delineated mechanistic response cascades [211, 215–218]	Some studies argue that it remains uncertain whether lactylation is a major driver or merely a minor regulator, and emphasize that only by precisely manipulating lactylation under pathophysiologically relevant conditions can one determine whether it is a causal node or a secondary consequence of high-lactate metabolism [219]
Does lactylation have a complete "writer-reader-eraser" system?^a^	"eraser": HDAC1/HDAC2/HDAC3 [209], SIRT1/SIRT3 [220, 221], SIRT2 [222], SIRT6 [223]	The system is not yet closed. Many questions remain unresolved regarding the identification of regulatory enzymes, site-specific substrates, and their functional mechanisms
	"writer":KAT2A (GCN5) [224, 225], ACSS2 [224, 225], GTPSCS [226], p300/CBP [226–229], HDAC1/HDAC2/HDAC3 (reverse-catalytic writers) [230], AARS1 [231], AARS2 [231], KAT2B [232, 233], HBO1 [233], KAT8 [234], HAT1 [235], NPM3 [236], HDAC6 [237]	
	"reader": DPF2 [218], TRIM33 [238], Brg1 [239]	
Who primarily "writes" lactylation?	Nuclear GTPSCS can function as a lactyl-CoA synthetase and cooperates with p300 to promote histone lactylation [226] The p300/CBP + lactyl-CoA pathway [240] AARS1 can directly utilize lactate and ATP to catalyze protein lactylation [241]	(1) Intracellular lactyl-CoA levels are far lower than those of acetyl-CoA, raising doubts as to whether p300 is the major lactyltransferase in vivo [241] (2) HDAC1/2/3 can directly catalyze Kla formation, and Kla abundance can be decoupled from lactyl-CoA levels [230]
Is site identification reliable?	The lactyllysine cyclic immonium ion (CycIm) can serve as a more reliable marker for indicating lysine lactylation [242]	The identification of "many sites" does not mean that every site has been validated with high confidence or shown to be functionally meaningful. Incomplete site-specific analyses and the lack of precise tools remain central limitations [243, 244]
Can lactylation serve as a therapeutic target?	Many studies related to metabolism and the immune microenvironment support lactylation as a biomarker and therapeutic target [244–246]	Challenges remain: (1) How can site-specific regulation be achieved without perturbing global lactate metabolism [247]? (2) There is compensatory crosstalk with other PTM networks [244]

^a^ACSS2 and GTPSCS are lactyl-CoA synthetases that form complexes with KAT2A (GCN5) and p300, respectively, thereby enhancing lactylation

In BMECs, lysine lactylation is localized predominantly in the nucleus [70], suggesting that lactate may participate directly in the epigenetic regulation of gene transcription. However, this evidence remains correlative, and the molecular basis, dynamic regulation, and functional significance of lactylation in ruminants remain unclear. A key unresolved question is whether lactylation in ruminants is driven mainly by D-lactate through non-enzymatic processes or by L-lactate through enzyme-dependent pathways. In non-ruminant systems, K_L-la_ is generally considered the dominant form of histone lactylation and is installed by specific enzymes [47], whereas K_D-la_ appears to arise independently of classical enzymatic machinery and may be more common on cytoplasmic proteins [212].

Evidence from ruminants, however, suggests a more complex scenario. Exogenous D-lactate has been shown to induce H3K18 lactylation in BMECs, and this effect is abolished by p300/CBP inhibition [49], seemingly at odds with the prevailing view that p300/CBP primarily catalyzes L-lactylation [248]. One possible explanation is that, in ruminants, enzyme-mediated L-lactylation remains the principal route linking metabolic state to transcriptional regulation, whereas D-lactate, which accumulates under pathological conditions such as acute ruminal acidosis, functions mainly as a signal of metabolic disruption. Rather than serving directly as the enzymatic substrate, D-lactate may indirectly enhance canonical L-lactate-dependent lactylation by altering intracellular lactate abundance, energy status, or redox balance. In this way, severe fermentation disturbances in the digestive tract may be translated into persistent epigenetic reprogramming in distant organs such as the mammary gland. Whether ruminants possess a distinct D-lactate-specific modification system remains an important question for future study.

### Lactate as a potential precursor for lactylation in ruminants

A key question in lactylation research is whether its precursors, lactate or lactyl-CoA, can enter the nucleus. In ruminants, direct evidence remains limited; therefore, current inferences must rely partly on findings from other experimental models.

As discussed above, negative energy balance in ruminants is accompanied by adipose mobilization, reduced insulin sensitivity along the adipose tissue-liver-skeletal muscle axis, and widespread reprogramming of lactate- and glycolysis-related fluxes. In mammalian cells, lactate dehydrogenase (LDH) catalyzes the production of L-lactate almost exclusively [249]. Accordingly, hyperlactatemia under these conditions arises mainly from metabolic reprogramming, and the accumulated lactate consists predominantly of endogenously produced, systemically distributed L-lactate [110, 250, 251]. As the principal circulating lactate isoform in mammals, L-lactate is a highly bioavailable metabolic intermediate that is efficiently transported between tissues via monocarboxylate transporters and participates in oxidative metabolism, metabolic signaling, and Cori cycle recycling [15, 252–254]. Notably, lactyl-CoA derived from L-lactate is considered the most direct precursor of K_L-la_ [47, 213]. Isotope-tracing and nuclear purification studies further suggest that cytoplasmic lactate can enter the nucleus and participate in intranuclear metabolism [255]. Current evidence favors a model in which lactyl-CoA is generated locally within the nucleus, likely through enzymes such as nuclear ACSS2 or GTPSCS, and is then utilized by lactylation-related complexes, including KAT2A and p300, to promote histone lactylation [225, 226]. Thus, local activation of nuclear L-lactate, rather than large-scale import of preformed cytoplasmic lactyl-CoA, is currently considered the more plausible source of lactylation substrates, although the relative contribution of these pathways may vary across cell types, metabolic states, and disease contexts.

By contrast, D-lactate arises mainly from aberrant bacterial fermentation. In ruminants, excessive grain and carbohydrate intake disrupts digestive fermentation, making the ruminal microbiota a major determinant of D-lactate production. This is particularly evident in SARA, where surges of gastrointestinal derived D-lactate may contribute to systemic inflammation. However, compounds absorbed through the portal circulation and exposed directly to hepatic metabolism generally show low systemic bioavailability [256]. Accordingly, much of the D-lactate generated in the rumen is likely to undergo substantial hepatic first-pass metabolism, thereby limiting the amount that reaches the systemic circulation [257]. Nevertheless, the possibility that rumen-derived D-lactate contributes to lactylation cannot be excluded. In dairy cow models of SARA, elevated lactate concentrations in plasma and mammary tissue are accompanied by increased Pan-Kla and H3K18la levels, together with upregulated MCT1, p300/CBP, and LDH expression [70, 85]. Although these studies did not distinguish lactate stereoisomers, SARA is typically associated with increased D-lactate, providing indirect support for its possible contribution. More direct functional evidence comes from BMECs, in which exogenous sodium D-lactate increases intracellular lactate levels and H3K18la, while enhancing NF-kB signaling and inflammatory cytokine production through a p300/CBP-dependent mechanism [49, 70]. These findings indicate that D-lactate can be utilized by mammary epithelial cells and is capable of driving histone lactylation. Thus, D-lactate may also serve as a potential precursor for lactylation in ruminants. However, its effective concentration in target tissues, nuclear accessibility, and quantitative contribution relative to L-lactate remain unresolved.

### Potential roles of lactylation in ruminants

The preceding sections have summarized the classical biological functions of lactate. Although these mechanisms provide a compelling framework for understanding the immediate effects of lactate as a stress-associated signaling molecule, they do not fully explain certain persistent phenomena observed in ruminant production. For example, why does SARA predispose animals to chronic mastitis? Likewise, how can the metabolic disturbances triggered by periparturient NEB exert a "memory effect", continuing to impair production performance even after the initial stressor has been removed? These observations suggest that lactate may not merely induce transient changes in cellular state through conventional signaling pathways, but may also reprogram gene expression through more profound and durable mechanisms. In this context, histone lactylation has been identified in diverse cell types and is increasingly recognized as an important component of the epigenetic regulatory network [47]. Consistently, immunofluorescence analyses in mammalian embryos have demonstrated the widespread presence of lactylation within the nuclear genome of oocytes and preimplantation embryos [258]. During early bovine embryonic development, Pan-Kla, H3K9la, and H3K18la levels undergo dynamic changes [259]. In addition, either increased or decreased histone lactylation inhibits bovine embryonic genome activation, thereby reducing early embryonic developmental efficiency and the capacity for the first lineage differentiation of blastocysts [259]. Moreover, alterations in lactylation are closely associated with changes in embryonic development and gene expression [258]. More direct mechanistic evidence was subsequently provided by a recent study showing that lactate regulates major zygotic genome activation through H3K18lac, thereby supporting a functional role of histone lactylation in embryonic gene regulation [260]. In addition, lipid peroxidation has been reported to induce atherosclerosis through the upregulation of lactate-dependent H3K18la [228], whereas aerobic fermentation-induced histone lactylation promotes autophagy in colorectal cancer cells by enhancing the transcription of Rubicon-like autophagy enhancer [261]. Together, these findings support the view that histone lactylation can regulate gene expression and thereby influence cellular function.

Emerging evidence further indicates that lactylation is not restricted to histones. Wang et al. [262] first identified pyruvate kinase M2 (PKM2) as a non-histone lactylation substrate and demonstrated that lactylation enhances its pyruvate kinase activity. They further showed that lactate, through PKM2 activation, suppresses glycolysis and promotes the transition of pro-inflammatory macrophages toward a reparative phenotype [262]. In addition, Chen [227] revealed that lactylation of the DNA double-strand break sensor MRE11 plays an important role in the metabolic regulation of homologous recombination repair. These observations indicate that non-histone protein lactylation may also alter the structure and function of target proteins [263]. Collectively, these studies provide broader support for lactylation as a regulatory mechanism linking metabolism with transcriptional control, protein activity, and cellular homeostasis.

In ruminants, however, current evidence for lactylation-dependent regulation remains focused mainly on H3K18la. In dairy cows under SARA, elevated lactate in mammary vein plasma and mammary gland tissue was accompanied by increased total lactylation, H3K18la, p300, and MCT1 expression in mammary tissues, together with enhanced inflammatory gene expression and activation of the TLR4/NF-κB pathway, suggesting an association between mammary gland lactylation and inflammatory responses in vivo [70]. In BMECs, LPS or exogenous D-sodium lactate treatment is accompanied by increased intracellular lactate and H3K18la, together with activation of the NF-κB signaling pathway; inhibition of p300/CBP by C646 reduces H3K18la levels and attenuates inflammatory responses, supporting the participation of p300/CBP-mediated H3K18la in this process [49]. Nevertheless, the precise downstream target genes directly regulated by histone lactylation in this model remain to be fully defined. In sheep, conceptus-derived lactate induces endometrial H3K18la, and both in vitro and in vivo evidence supports a role for lactate-induced histone lactylation in uterine remodeling by regulating redox homeostasis, apoptotic balance, and implantation-related endometrial receptivity [264]. Table 3 presents a summary of the current research on lactylation in ruminants.

**Table 3:** Current evidence for lactylation in ruminants

Species	Tissue	Sources of lactate	Site	Reference
Bovine	BMECs	Endogenous lactate (produced by LPS-stimulated cells) and exogenous lactate (sodium D-lactate)	H3K18la	[49]
Bovine	Mammary gland	Endogenous lactate (produced by high-concentrate diet)	H3K18la	[70]
Bovine	SCNT embryos	Exogenous lactate (the original IVC medium containing lactate)	H3K18la	[165]
Bovine	In vitro fertilized embryos	GSKA, an LDHA inhibitor, was used to reduce intracellular lactate production. Sodium lactate served as an exogenous lactate donor. β-nicotinamide mononucleotide (NMN) rescued the GSKA-induced decrease in lactylation	H3K9la, H3K18la	[259]
Sheep	Pregnant sheep endometrial cancer cell line	Endogenous lactate (during the period of embryo implantation, the glycolysis of sheep is enhanced, generating a large amount of lactate)	H3K18la	[264]

The evidence summarized in this table should be interpreted with caution. In many ruminant studies, the available data mainly indicate an association between lactate accumulation and changes in histone lactylation, but lack downstream validation, such as identification of specific target genes, chromatin/promoter occupancy, functional intervention assays, or direct demonstration of lactylation-dependent regulation. Therefore, these findings primarily support a correlation between lactate and histone lactylation rather than a definitively established lactylation-mediated mechanism

Additional animal studies provide more direct support for lactylation-dependent regulation. In DON-induced ovarian injury in piglets, DON reduces lactate production and specifically decreases H3K18la, whereas lactate supplementation restores H3K18la and alleviates ferroptosis-related injury [265]. More importantly, promoter-level analyses further linked H3K18la with downstream transcriptional regulation of genes such as STEAP3, providing stronger support for a lactylation-dependent mechanism in this model [265]. Similarly, a multispecies sleep-deprivation study showed that elevated glycolysis and lactate accumulation promoted H3K18la and transcriptionally activated RORα, thereby identifying a relatively complete lactate-H3K18la- RORα signaling cascade across mice, zebrafish, and pigs [42]. Taken together, these findings indicate that lactylation may represent an important epigenetic mechanism by which lactate influences physiological and pathological processes. Nevertheless, the strength of evidence varies across studies. Only those phenotypes supported by convergent evidence, including changes in lactylation marks, modifying enzymes, promoter occupancy or target-gene expression, and functional intervention experiments, should be interpreted as lactylation-dependent regulation. By contrast, observations based solely on lactate accumulation together with phenotypic alteration are more appropriately described as lactate-associated rather than definitively lactylation-driven.

Despite these advances, research on lactylation in ruminants remains at an early stage. Current evidence is largely restricted to H3K18la in the bovine mammary gland and the ovine endometrium, whereas other lactylation sites, non-histone lactylation, and genome-wide target identification remain insufficiently explored. This limitation is particularly noteworthy because ruminants experience unique lactate-related physiological and pathological conditions, such as SARA, negative energy balance, oxidative stress and embryo implantation, all of which may provide biologically relevant settings for lactylation-dependent regulation. Therefore, elucidating the molecular basis and biological significance of lactylation in ruminants will be essential for understanding the links among lactate metabolism, epigenetic regulation, inflammation, and reproduction.

## Conclusions and future perspectives

Taken together, lactate participates in a wide range of physiological and pathological processes in ruminants. Conditions such as subacute ruminal acidosis, ketosis, mastitis, and other forms of oxidative stress in dairy cows are frequently accompanied by lactate accumulation [136, 137]. Consequently, this lactate-enriched acidic microenvironment may provide favorable conditions for the occurrence of lactylation. In this context, histone lactylation appears to regulate ruminant metabolism in close association with anaerobic glycolysis. Accumulating evidence indicates that histone lactylation plays a pivotal role in both physiological and pathological settings [47]. Nevertheless, compared with other epigenetic modifications, many aspects of histone lactylation remain poorly understood, and a substantial knowledge gap persists regarding its specific roles and mechanisms in ruminants.

Several key questions remain unresolved, for example: (a) It is still unclear whether histone lactylation is an inevitable consequence of lactate accumulation or a precisely regulated process under spatiotemporal control. (b) Direct evidence for the regulatory mechanisms governing lactylation in ruminants is still lacking. In particular, it remains unknown whether histone or non-histone lactylation plays the dominant role in ruminant regulation, and whether these two forms are functionally interconnected or potentially interconvertible. (c) The specific enzymes responsible for the writing, reading, and erasing of lactylation in ruminants have yet to be identified. (d) How histone lactylation interacts with other post-translational modifications to coordinately regulate gene transcription also remains to be clarified. (e) In addition, the non-histone proteins subject to lactylation in ruminants remain largely unknown. Therefore, systematic identification of lactylation sites and the construction of a comprehensive lactylation map in ruminants would substantially advance this field. Moreover, elucidating how diverse metabolic stresses encountered in ruminant production drive lactylation through lactate-dependent mechanisms will be of considerable importance for safeguarding ruminant health.

## Data Availability

No datasets were generated or analysed during the current study.

## Abbreviations

ARA: Acute ruminal acidosis
bFLS: Bovine fibroblast-like synoviocytes
BMECs: Bovine mammary epithelial cells
DMI: Dry matter intake
ET: Extracellular trap
G-CSF: Granulocyte colony-stimulating factor
GLO2: Glyoxalase II
HIF-1α: Hypoxia-inducible factor-1α
IR: Insulin resistance
Kce: Nε-(carboxyethyl)lysine
K_D-la_: D-Lactoylation
Kla: Lysine lactylation
K_L-la_: L-Lactylation
LDH: Lactate dehydrogenase
LGSH: S-lactoylglutathione
LPS: Lipopolysaccharides
MCT: Monocarboxylate transporters
MGO: Methylglyoxal
NEB: Negative energy balance
NEFAs: Non-esterified fatty acids
NET: Neutrophil extracellular trap
PDH: Pyruvate dehydrogenase
PKM2: Pyruvate kinase M2
PMNs: Polymorphonuclear leukocytes
PPARs: Peroxisome proliferator-activated receptors
ROS: Reactive oxygen species
SARA: Subacute ruminal acidosis
SCNT: Somatic cell nuclear transfer
sMCTs: Sodium-coupled monocarboxylate transporters
TCA: Tricarboxylic acid
